# The motor skills and sensory processing abilities associated with idiopathic toe walking gait

**DOI:** 10.1186/1757-1146-6-S1-O38

**Published:** 2013-05-31

**Authors:** Cylie Williams, Paul Tinley, Michael Curtin, Sharon Nielsen

**Affiliations:** 1 Allied Health Research Unit, Southern Health, Cheltenham, VIC, 3192, Australia; 2 Department of Physiotherapy, Monash University, Frankston, VIC, 3199, Australia; 3 School of Podiatry, Charles Sturt University, Albury, NSW, 2460, Australia; 4 School of Occupational Therapy, Charles Sturt University, Albury, NSW, 2460, Australia; 5 Quantitative Consulting Unit, Charles Sturt University, Wagga Wagga, NSW, 2678, Australia

## Background

This study aimed to investigate differences between the motor skills and sensory processing abilities of children between the ages of four and eight years with and without an idiopathic toe walking (ITW) gait.

## Methods

Children in each cohort were tested with the following norm referenced assessments:

1. Bruininks-Oseretsky Test of Motor Proficiency 2nd edition (BOT-2)

2. The Sensory Profile (SP)

3. Six subtests of the Sensory Integration and Praxis Tests (SIPT)

4. Vibration Perception Threshold (VPT)

## Results

Sixty children participated in the study, 30 within each cohort. Those with an ITW gait were found to have different SP quadrant scores (p=0.002), poorer performance on the BOT-2 (p=<.001), a lower VPT (p=.001) and poorer performance on the Standing Walking Balance subtest of the SIPT (p=0.047) compared with peers.

## Conclusion

While the results did not identify a causative factor for an ITW gait, they do suggest that the toe walking gait may not be idiopathic in nature. The results of this research highlight the importance of a fuller assessment of the toe walking child compared to that traditionally conducted by podiatrists, and suggest that multiple strategies may be required to manage this gait style.

